# Multigenerational exposure of Ag materials (nano and salt) in soil – environmental hazards in *Enchytraeus crypticus* (Oligochaeta)†

**DOI:** 10.1039/d3na00487b

**Published:** 2023-11-22

**Authors:** Fátima C. F. Santos, Rudo A. Verweij, Amadeu M. V. M. Soares, Janeck J. Scott-Fordsmand, Cornelis A. M. van Gestel, Mónica J. B. Amorim

**Affiliations:** a Department of Biology & CESAM, University of Aveiro 3810-193 Aveiro Portugal mjamorim@ua.pt; b Amsterdam Institute for Life and Environment (A-LIFE), Faculty of Science, Vrije Universiteit Amsterdam De Boelelaan 1085 1081 HV Amsterdam The Netherlands; c Department of Ecoscience, Aarhus University C. F. Møllers Alle 4 DK-8000 Aarhus Denmark

## Abstract

Because of its properties, silver is among the most used metals both as salt and as nanomaterials (NMs), hence reaching the environment. Multigenerational (MG) exposure testing is scarce, and especially so for NMs and soil invertebrates. In this study the MG effects of Ag NMs (Ag NM300K) and Ag salt (AgNO_3_) were assessed, using *Enchytraeus crypticus* in LUFA 2.2 soil. Survival, reproduction and internal Ag concentration in the animals were measured throughout 7 generations (5 generations (F0–F4) in spiked soil plus 2 (F5–F6) in clean soil) exposed to sublethal concentrations corresponding to the reproduction EC_10_ and EC_50_ obtained in standard toxicity tests (45 and 60 mg Ag per kg soil DW for AgNO_3_; 20 and 60 mg Ag per kg soil DW for Ag NM300K). MG exposure caused a dose-related decrease in reproduction for both Ag forms. Ag uptake peaked in the F1 (64 days) for AgNO_3_ and F2 (96 days) for Ag NM300K, after which it decreased. In agreement with toxicokinetic studies, a maximum body Ag concentration was reached (20 mg Ag per kg body DW (AgNO_3_) and 70 mg Ag per kg body DW (Ag NM300K)) and after which detoxification mechanisms seem to be activated with elimination of Ag accompanied by a decrease in reproduction. Transfer to clean soil allowed Ag to be (fully) eliminated from the animals. This MG study confirmed the effects determined in standard reproduction toxicity tests but further allowed to monitor the dynamics between exposure and effects of the Ag materials, and how the animals seem to cope with Ag for 7 generations by compensating between detoxification and reproductive output.

## Abstract 2

Ag NM300K and AgNO_3_ kinetics showed how animals handle Ag exposure for 7 generations, compensating between detoxification and reproduction. Internal Ag peaked (F1 for AgNO_3_ (20 mg Ag) and F2 for Ag NM300K (70 mg Ag), after which detoxification co-occurred with decrease in reproduction.

## Graphical abstract

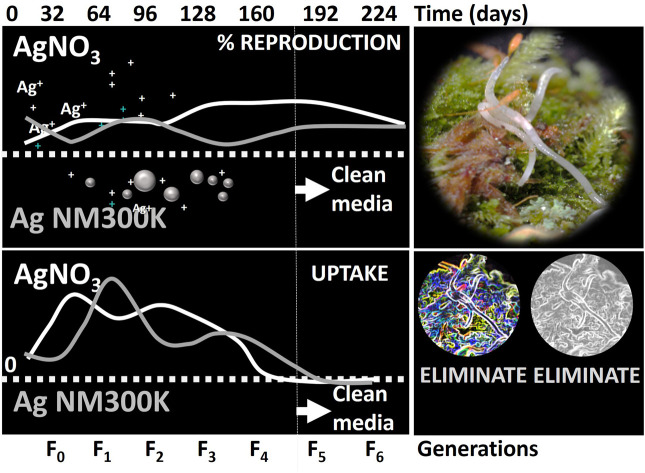

## Introduction

Silver (Ag) is one of the most used metals in the nanomaterial (NM) form, with an estimated total production in the European Union (EU) of about 50 tons in 2014.^1,2^ Due to its antimicrobial activity properties, Ag is applied in a wide range of products, both in the salt and nano form, *e.g.* hospital supplies, clothing, daily care products, food packaging, water disinfectants, refrigerators.^3–7^ Silver is retained in wastewater treatment plants and the application of sewage sludge to soil is one of the largest sources of entry in the terrestrial environment, which acts as a sink for this element.^1,8^ The increasing use of Ag in various applications leads to its increasing entry into the soil environment.^3,8,9^ Soil organisms can be exposed to increasing levels of Ag, hence concerns on its potential adverse effects increase.^10–13^ Silver NM toxicity has been shown in soil invertebrates, even at low concentrations, *e.g.*, in *Enchytraeus crypticus*,^14^*Lumbricus rubellus*,^15^*Eisenia fetida*,^16^*Eisenia andrei*, *Folsomia candida*,^17^ and *Porcellionides pruinosus*.^18^ The exposure period in standard toxicity tests commonly ranges from 21 to 28 days (enchytraeids, collembolans) to 56 days (earthworms) to assess chronic effects like on the reproduction of these soil invertebrates. Experience with NMs has often shown that effects occur at longer exposure times.^19–21^ For instance, results from a full life cycle test with *E. crypticus*,^14^ with exposure to silver (AgNO_3_ and Ag NM300K) over a longer period of time, allowed to identify effects of the two Ag forms which could not be shown *via* the standard test alone. It has been long recognized that longer-term testing is needed, *e.g.*, exposure to a low concentration of a contaminant for a longer time can be even more hazardous than a short-term exposure to higher concentrations.^22^ Multigenerational (MG)^23^ exposure occurs when chemicals are present at lower concentrations in the environment, often when highly persistent. Moreover, it has been shown that for NMs special attention should be given to lower concentrations, where highest hazards can be observed, as shown *e.g.* for AgNMs in *E. crypticus*^14^ which showed a non-monotonic dose–response relationship. Cumulative damage over generations (like due to oxidative stress that can cause DNA damage) may only become evident after several generations.^23^ An MG approach with longer exposure durations and more sampling points carries more potential to both detect and interpret effects.^24^

MG testing is scarce, especially for soil organisms, although some studies have been performed in recent years.^20,25,26^ For both *E. crypticus* and *F. candida* a comparable test design has been developed, with exposure to spiked soil for 4 consecutive generations followed by incubation in clean soil for 2 generations, which allows to further analyze potential transgenerational impacts of (chemical) stressors. A multigenerational exposure is particularly relevant when studying persistent materials as is also the case for many NMs. Hence, it was aimed to investigate the effects of prolonged multigenerational exposure to Ag NM in soil-dwelling organisms, using *E. crypticus* as a model species. Enchytraeids have a key role in soil ecosystems, they promote organic matter decomposition and aeration of the soil. In this study the effects of an MG exposure (5 generations exposed + 2 generations in clean soil) to Ag NM300K and AgNO_3_ were investigated, using the soil model organism *E. crypticus*. Survival and reproduction were assessed throughout, and Ag concentrations were measured, both in the animals and the soil, following exposure to sublethal Ag concentrations corresponding with the EC_10_ and EC_50_ for effects on reproduction in standard toxicity tests. The reference JRC Ag NMs^27^ were selected given the vast characterization and prior knowledge of their hazards to *E. crypticus*, covering various endpoints, namely the ones in the standard reproduction and the full life cycle toxicity tests (hatching, growth, maturity, survival, reproduction),^14^ in avoidance,^28^ gene expression,^29^ toxicokinetics^30^ and toxicodynamics studies.^31^

## Materials and methods

### Test organisms

*Enchytraeus crypticus* (Oligochaeta: Enchytraeidae) were maintained in cultures in agar media, consisting of Bacti–Agar medium (Oxoid, Agar No. 1) and a sterilized mixture of 4 different salt solutions at final concentrations of 0.08 mM KCl, 2 mM CaCl_2_·2H_2_O, 1 mM MgSO_4_, and 0.75 mM NaHCO_3_. Age-synchronized cultures were prepared as described in ref. 32. In short, adult organisms with well-developed clitellum were transferred to agar plates for cocoon laying, after which the cocoons (1–2 days old) were transferred to new agar and allowed to hatch. Juveniles of 19–21 days old after cocoon laying were used for the parental exposures in this study.

### Test soil

The natural LUFA 2.2 standard soil (Speyer, Germany) was used. The main characteristics were (as provided by the supplier): pH (0.01 M CaCl_2_) = 5.6, organic carbon = 1.77%, cation exchange capacity = 8.5 cmol_c_ per kg, maximum water holding capacity (WHCmax) = 43.3%, and grain size distribution of 10.6% clay (<0.002 mm), 15.0% silt (0.002–0.05 mm), and 74.4% sand (0.05–2.0 mm).

### Test materials, characterisation and spiking procedures

Silver nitrate (AgNO_3_, purity >99%, Sigma-Aldrich) and the reference silver nanomaterial Ag NM300K, from the European Commission Joint Research Centre (JRC), were used. Ag NM300K is fully characterized, for full details see.^27^ Ag NM300K are spherical, consisting of a colloidal dispersion with a nominal silver content of 10.2% w/w, dispersed in 4% w/w of polyoxyethylene glycerol trioleate and polyoxyethylene sorbitan monolaurate (Tween 20). Approximately >99% of the particles have a nominal size of 15 nm. Transmission electron microscopy (TEM) shows a size of 17 ± 8 nm and smaller nanoparticles of *ca.* 5 nm were also present.

The tested concentrations were 0–45–60 mg Ag per kg soil DW for AgNO_3_ and 0–20–60 mg Ag per kg soil DW for Ag NM300K, selected within the sub-lethal range, based on the EC_10_ and EC_50_ for effects on reproduction in standard enchytraeid toxicity tests.^14^

Aqueous solutions of AgNO_3_ and Ag NM300K were prepared by diluting with water to the required concentrations. Spiking was performed by mixing the aqueous solutions with pre-moistened soil batches, which were then thoroughly mixed to obtain a homogeneous distribution and split into replicates. For the highest concentration of Ag NM300K (60 mg Ag per kg soil DW), spiking was done replicate per replicate. Soil moisture content was adjusted to 50% of the WHCmax. Soil was freshly spiked 1 day prior to the start of the exposure of each generation. Soil (3 g per treatment) was collected for Ag concentration quantification at the beginning of the exposure of each generation, dried at 40 °C for 48 hours and stored.

### Experimental procedure

The standard OECD test guideline 220 (ref. 33) was followed with adaptations as described in ref. 20. In short, 40 juveniles (19–21 days old) were used per replicate to start the test. Test vessels were prepared containing 40 g of moist soil and food supply (ground oats: 36 ± 1 mg). Test duration was 32 days per generation and tests ran at 20 °C, with a 16 : 8 h photoperiod. The test design included a total of 7 generations with a total test duration of 224 days: 5 generations in spiked soil (F0–F4) plus 2 generations in clean soil (F5 and F6) to evaluate recovery. Six replicates were used for the control and the EC_10_, and 10 replicates for the EC_50_ exposure (the extra replicates were used to compensate for mortality and ensure enough animals would be available to start a next generation). Water and food were replenished weekly. At the end of each generation, organisms were collected directly from the soil and placed in ISO water^34^ for a short period of time to clean from soil particles. Juveniles (*n* = 40 per replicate) of medium size were collected and transferred to freshly spiked soil for the next generation exposure. Adults (*n* = 8, per treatment) were collected for Ag quantification, kept in ISO water^34^ for 12 hours to purge the gut from soil particles, then blotted dry on filter paper, stored individually and frozen at −20 °C. The remaining soil containing organisms was fixated with 96% ethanol and stained with Bengal rose (1% in ethanol) to count the total number of animals. The replicates were sieved through 3 decreasing pore size meshes (1.6, 0.5 and 0.3 mm) to help separating most of the soil from the organisms and facilitate counting. The reproduction and survival were assessed by counting the juveniles and adults with the help of a stereo microscope.

### Ag measurement

Concentration of Ag was measured in animals and soil using graphite furnace atomic absorption spectrometry (AAS; PinAAcle 900Z, PerkinElmer, Singapore) and flame AAS (AAnalyst 100; PerkinElmer; Germany), respectively. Sample preparation was as follows: organisms were freeze-dried for 24 hours, weighted individually and digested with 300 μL of a mixture of HNO_3_ (Fischer Scientific OPTIMA Grade, Loughborough, UK) and HClO_4_ (70%; J.T.Baker (Avantor) Ultrex Ultra-Pure, Radnor, PA, USA) in a block heater (Techne Dri-Block Heater, Staffordshire, UK) using a heating ramp ranging from 85 to 180 °C. After all acid was evaporated, the left residue was dissolved in 300 (or 500) μL of 1 M HCl and Ag concentrations in the digests were measured by AAS. Limit of detection (LOD) for Ag was 0.003 mg kg^−1^ dry body weight, which is well within the experimental design requirements.

To determine total Ag concentrations in the test soil, approx. 130 mg dry sample from each replicate of each sampling day were digested using 2 ml of a destruction mixture of HNO_3_ (65%, Sigma-Aldrich) and HCl (37%, Sigma-Aldrich) (1 : 4 v/v), in Teflon containers that were closed tightly and heated at 140 °C for 7 hours. After cooling, 8 ml of deionized water was added, and the Ag concentrations measured by flame AAS. Certified reference material LGC 6181 was included in the analysis; mean (±SD; *n* = 2) silver concentrations measured in the reference material were 85.7 ± 0.69% of the certified values; all measured soil concentrations were corrected for this recovery. LOD for Ag analysis in soil samples was 0.003 mg Ag per kg dry soil, which is well within the experimental design requirements.

### Data analysis

One-way analysis of variance (ANOVA) followed by Dunnett's comparison post-hoc test (*p* ≤ 0.05) was used to assess the significance of differences between F0 and the other generations within each treatment.^35^

## Results

The soil pH (0.01 M CaCl_2_) did not significantly change during the test duration and with Ag treatment (Table S1†), being (average ± standard error) 6.0 ± 0.07 and 6.0 ± 0.05 for AgNO_3_ and 6.1 ± 0.02 and 6.1 ± 0.04 for Ag NM300K at the beginning and end of the tests, respectively.

Measured Ag concentrations in the spiked soils ranged between 73.0 and 113% of nominal ones (Table S2†), with average of 81.1 and 95.1% for AgNO_3_ at the EC_10_ and EC_50_ level, respectively. For Ag NM300K, Ag recovery ranged between 79.3 and 114%, with one outlier of 251% at the EC_10_ level in F2 (Table S2†). This resulted in average recoveries of 127 and 91.8% at the EC_10_ and EC_50_, respectively, with the high average recovery at the EC_10_ explained from the outlier at the F2.

The validity criteria for control performance of the test organisms, as defined by the OECD test guideline 220,^33^ were fulfilled. Control adult mortality was well below 20% (2–11% for AgNO_3_ and 4–7% for Ag NM300K) (Table S3†); juvenile numbers in the controls were always far above the minimum of 25 per 10 animals, ranging between 2700 and 5250 for the AgNO_3_ test and between 2473 and 4460 for the test with Ag NM300K (Table S3†). The coefficient of variation of juvenile numbers was always <20% (Table S3†), ranging between 6 and 18% for AgNO_3_ and between 9 and 20% for the NM300K exposures.

**Fig. 1:**
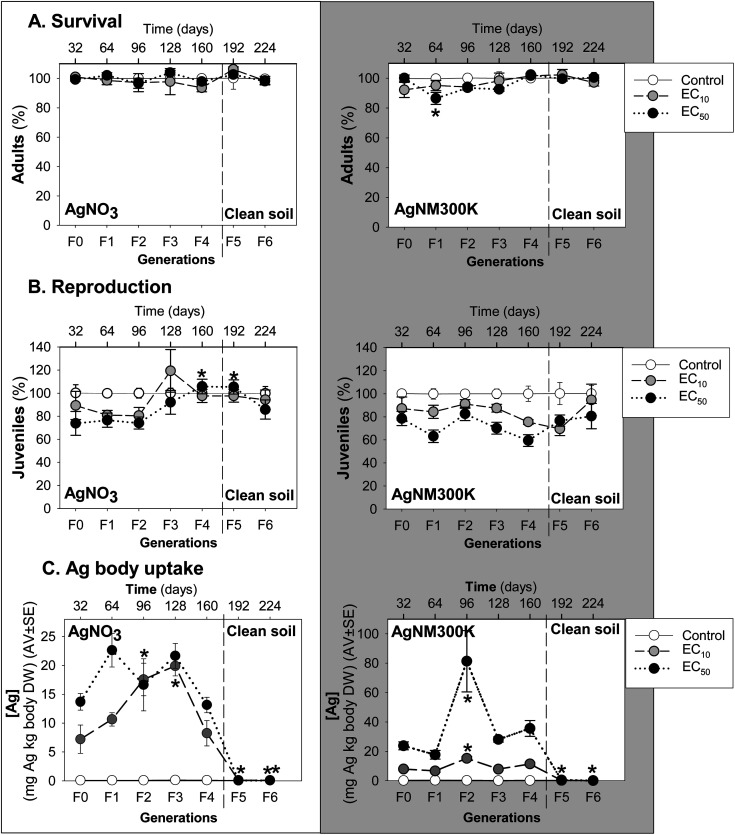
Results of the multigenerational test with *Enchytraeus crypticus* exposed for five generations (F0–F4) to AgNO_3_ (0–45–60 mg Ag per kg soil DW) and Ag NM300K (0–20–60 mg Ag per kg soil DW) in LUFA 2.2 natural soil followed by two generations in clean soil (F5-6). Measured endpoints are (A) survival, (B) reproduction, and (C) internal Ag concentrations in surviving animals. Results for survival and reproduction are expressed as % of the control. All values are presented as average ± standard error (AV ± SE), and exposure concentrations are indicated as effect levels (control, EC_10_ and EC_50_). *p < 0.05 (Dunnett's test for differences between parental generation (F0/F1) and the other generations).

Enchytraeid survival (Fig. 1A) was not significantly affected by MG exposure, except for the decrease in the Ag NM300K exposure in the F1 at the EC_50_ level.

MG exposure to AgNO_3_ caused a small dose-related decrease in enchytraeid reproduction up to F2, followed by an increase in F3, most pronounced at the EC_10_ level (higher than control) (Fig. 1B). This was followed by a decrease to juvenile numbers similar to the control in F4, a trend continuing in the last two generations in clean soils (F5-6).

For Ag NM300K MG exposure, a dose-related decrease occurred in reproduction up to F1, “interrupted” by an increase in F2, and followed by a decrease until F4 (Fig. 1B). After transfer to clean soil, increasing recovery was observed throughout F5 to F6.

The internal Ag concentrations measured in the surviving animals (Fig. 1C) of the exposure to AgNO_3_ showed that Ag uptake increased from F0 to F3, from 7 mg Ag per kg body DW (at the EC_10_) and 13 mg Ag per kg body DW (at the EC_50_) up to a maximum of 22 mg Ag per kg body DW (at both EC_10_ and EC_50_), after which it decreased in F4 to concentrations equivalent to F0. After transferring to clean soil (F5 and F6) the Ag concentration in the animals was negligible.

For Ag NM300K, a dose-related increase of Ag body concentrations was seen in F0 and F1 (8 and 24 mg Ag per kg body DW for EC_10_ and EC_50_, respectively) followed by a peak in F2, reaching a maximum of 80 mg Ag per kg body DW in the exposure to 60 mg Ag NM300K per kg soil DW (EC_50_). At the EC_10_ level, maximum body concentration in the F2 was about 20 mg Ag per kg body DW. This peak in the F2 was followed by a decrease of Ag body concentrations in F3 and F4 to values similar to F0–F1. After transfer to clean soil, Ag concentration in the animals was close to zero.

## Discussion

Exposure to sublethal reproduction EC_10_ and EC_50_ of AgNO_3_ and Ag NM300K did not affect long-term survival of *E. crypticus* in LUFA 2.2 soil. Similar MG studies with CoCl_2_ and WCCo NM^26^ and CuCl_2_ and CuO NM^20^ showed no major variations in the survival of *E. crypticus* also exposed to concentrations corresponding with the EC_10_ and EC_50_ for effects on reproduction in standard toxicity tests. This is not surprising since the exposure concentrations were sublethal but there could be an unknown accumulation of effects *via* MG exposure, as observed *e.g.* in *F*. *candida* exposed for multiple generations to phenanthrene.^36^ The reproduction response at F0/F1 was as expected based on the ECx determined previously in standard toxicity tests.^14^

A large variation was observed for the internal Ag concentrations in the enchytraeids, in particular for Ag NM300K in the F3. Such large variation has been reported previously, *e.g.*, by.^26,37–40^ Variability is naturally expected due to individual genetic variation, sexual reproduction, age, patchy soil exposure, to name some of the factors. Body Ag concentrations in the enchytraeids did not clearly explain toxicity, as no clear relationships were found between juvenile numbers produced in the different generations and body Ag concentrations in the adults. Nevertheless, the reproduction in the AgNO_3_ MG test at the EC_10_ level showed an increase in F3, which coincided with a higher Ag uptake while the reduction in juvenile numbers in the following generations also went along with a decrease in Ag body concentrations. For AgNM300K a similar pattern was seen but one generation earlier, in the F2, and with a less wide increase in reproduction with Ag body concentrations, especially for the EC_50_, after a peak in internal Ag concentration measured in F2 (80 mg Ag per kg body DW). Also in this case, the decrease in Ag body concentrations in the F3 and F4 coincided with a decrease in reproduction. This may perhaps indicate a trade-off in energy allocation between detoxification and reproduction, *i.e.*, more energy was spent on detoxification/elimination of Ag and hence there was less energy to reproduce. The MG effect of Ag NM300K was different from that of CuO NM^20^ which increased throughout the generations, while for Ag NM300K the initial increase was followed by a decrease in effect. For both Ag forms, transfer to clean soil allowed animals to recover reproduction to the control level.

Moreover, results of this MG study were in agreement with those of toxicokinetic studies, where a maximum uptake of 20 mg Ag per kg body DW and 70 mg Ag per kg body DW was observed after 14 days exposure to 45 mg AgNO_3_ per kg soil DW and 60 mg AgNM300K per kg soil DW, respectively.^38^ This might suggest that after reaching an internal Ag threshold, detoxification mechanisms were activated and Ag was more efficiently eliminated, co-occurring with a reduction in reproduction. A similar pattern was observed for exposure to CoCl_2_ where at F4 reproduction peaked for *E. crypticus* exposed to the EC_10_ and EC_50_, also corresponding with an increase in the uptake of Co, *i.e.*, internal Co concentration at F3.^26^ This trend is confirmed by a study where the exposure to CoCl_2_ was extended up to 56 days (equivalent to an additional generation or F2), which showed an increase in the impact at the population level,^41^ also after a peak in the internal Co concentration in the F1 (28 days exposure). For *E. crypticus*, MG exposure to CuCl_2_ at the EC_10_ and EC_50_ (ref. 41) also showed an increased reproduction from F2 to F5, although in this case we do not know if there was a relation with internal Cu concentration.

A similar MG exposure design with *F*. *candida* showed lower impacts, with no effects for CuO NM up to 6400 mg Cu per kg soil DW or for WCCo NM where effects on reproduction and survival only occurred from the F3 onwards, with EC_50_ values between 2400 and 5600 mg WCCo NM per kg soil DW.^25^ Nevertheless, expression of target genes was affected in all exposures and generations, and not always returned to control levels after transfer to clean soil. This indicates the different species sensitivity but also that the mechanisms were triggered even though without apparent phenotypic toxicity.

## Conclusions

The MG exposure of *E. crypticus* to AgNO_3_ and Ag NM300K showed that Ag uptake was dose-related and increased throughout generations, peaking in the F1 (64 days) for AgNO_3_ and F2 (96 days) for Ag NM300K, after which it decreased. In line with earlier toxicokinetic studies, a maximum body Ag concentration was reached (20 mg Ag (AgNO_3_) per kg body DW and 70 mg Ag (AgNM300K) per kg body DW), after which detoxification mechanisms seemed to be activated leading to elimination of Ag and co-occurring with a decreased reproduction. Transfer to clean soil allowed Ag concentrations in the animals to return to background levels. This MG study confirms the effects at the EC_10_ and EC_50_ levels of exposure to AgNO_3_ and Ag NM300K determined in standard reproduction toxicity tests. MG results allowed to monitor the dynamics between exposure and effects of Ag materials, and how the animals seem to cope with exposure throughout 7 generations by compensating between detoxification and reproductive output.

## Author contributions

FCFS: formal analysis, investigation, methodology, writing – original draft, RV: investigation, AMVM: conceptualization, CvG, JSF, MJBA: conceptualization, data curation, formal analysis, funding acquisition, resources, supervision, writing – original draft. All authors: write – review & editing.

## Conflicts of interest

There are no conflicts of interest to declare.

## Supplementary Material

NA-006-D3NA00487B-s001
