# Supplementary material for: Multigenerational exposure of Ag materials (nano and salt) in soil – environmental hazards in Enchytraeus crypticus (Oligochaeta)
Source: Nanoscale Adv. 2023 Nov 22;6(3):826–31. doi: 10.1039/d3na00487b (PMC10825909; doi:10.1039/d3na00487b)
Supplement: NA-006-D3NA00487B-s001 [file NA-006-D3NA00487B-s001.pdf]

Electronic Supplementary Material (ESI) for Nanoscale Advances.
This journal is © The Royal Society of Chemistry 2023

**Supplementary information**

# Multigenerational exposure of Ag materials (nano and salt) in soil – environmental hazards in *Enchytraeus crypticus* (Oligochaeta)

Fátima C.F. Santos[a], Rudo A. Verweij[b], Amadeu M.V.M. Soares[a], Janeck J. Scott-Fordsmand[c], Cornelis A.M. van Gestel[b], Mónica J.B. Amorim[a]*

[a]Department of Biology & CESAM, University of Aveiro, 3810-193 Aveiro, Portugal

[b]Amsterdam Institute for Life and Environment (A-LIFE), Faculty of Science, Vrije Universiteit Amsterdam, De Boelelaan 1085, 1081 HV Amsterdam, The Netherlands

[c] Department of Ecoscience, Aarhus University, C.F. Møllers Alle 4, DK-8000, Aarhus, Denmark

*Corresponding author: mjamorim@ua.pt

**Table S1**: Soil pH (0.01 M $CaCl_2$) measured at the start (t=0) and at the end (t=32) of each generation in a multigeneration test with *Enchytraeus crypticus* exposed in LUFA 2.2 soil spiked with $AgNO_3$ and Ag NM300K. F5 and F6 were exposed to clean soil.

| | pH ($CaCl_2$) | | | | | | | | | | | | | |
|---|---|---|---|---|---|---|---|---|---|---|---|---|---|---|
| Generation | F0 | | F1 | | F2 | | F3 | | F4 | | F5 | | F6 | |
| Time (days) | 0 | 32 | 0 | 32 | 0 | 32 | 0 | 32 | 0 | 32 | 0 | 32 | 0 | 32 |
| $AgNO_3$ (mg Ag/kg soil DW) | | | | | | | | | | | | | | |
| 0 | 6.3 | 6.1 | 6.1 | 5.9 | 6.1 | 5.8 | 5.9 | 5.4 | 6.0 | 5.8 | 5.9 | 5.9 | 6.0 | 5.6 |
| 45 | 5.7 | 6.2 | 5.9 | 6.2 | 6.1 | 5.8 | 5.7 | 6.1 | 5.9 | 6.0 | 6.1 | 6.0 | 6.0 | 5.8 |
| 60 | 5.6 | 6.1 | 5.7 | 6.3 | 6.0 | 6.1 | 5.8 | 6.0 | 5.8 | 6.2 | 6.1 | 6.1 | 6.1 | 5.9 |
| Ag NM300K (mg Ag/kg soil DW) | | | | | | | | | | | | | | |
| 0 | 5.8 | 5.6 | 6.0 | 6.0 | 6.0 | 6.0 | 6.0 | 6.3 | 6.2 | 5.9 | 6.1 | 5.7 | 6.0 | 6.0 |
| 20 | 5.8 | 6.1 | 6.0 | 6.3 | 6.1 | 6.3 | 6.1 | 6.2 | 6.1 | 6.1 | 6.1 | 6.0 | 6.1 | 6.2 |
| 60 | 5.8 | 6.1 | 6.1 | 6.1 | 6.0 | 6.1 | 6.0 | 6.2 | 6.1 | 6.1 | 6.2 | 6.1 | 6.1 | 6.1 |

**Table S2**: Measured total soil concentrations and internal Ag concentrations in the organisms (AV ± SE) measured at the end of each generation, so at days 32, 64, 96, 128, 160, 194 and 224 of the multigenerational tests with *Enchytraeus crypticus* in LUFA 2.2 soil spiked with $AgNO_3$ (45 and 60 mg Ag/kg soil DW) and Ag NM300K (20 and 60 mg Ag/kg soil DW). Also shown are the generation and effect level of exposure. [% Recovery]: recovery of Ag in the spiked soils compared to nominal concentrations of $AgNO_3$ and Ag NM300K. F5 and F6 were exposed to clean soil.

| **Gene-ration** | **Time** (days) | **Effect level** | **Measured total** (mg/ kg soil DW) [% recovery] | **Internal conc.** (mg Ag/kg body DW) | **Measured total** (mg/ kg soil DW) [% recovery] | **Internal conc.** (mg Ag/kg body DW) |
|---|---|---|---|---|---|---|
| | | | **$AgNO_3$** | | **Ag NM300K** | |
| **F0** | 32 | Control | 0 | 0.08±0.013 | 0.05±0.05 | 0.13±0.02 |
| | | $EC_{10}$ | 38.2±0.5 [84.9%] | 7.2±2.4 | 20.3±0.5 [102%] | 7.9±1.1 |
| | | $EC_{50}$ | 67.7±1.5 [113%] | 13.7±1.4 | 57±3.2 [95%] | 23.7±2.7 |
| **F1** | 64 | Control | 0.05±0.05 | 0.07±0.02 | 0.04 ± 0.04 | 0.20±0.03 |
| | | $EC_{10}$ | 33.0±0.2 [73.0%] | 10.6±1.2 | 17.1±0.1 [85.5%] | 6.6±0.9 |
| | | $EC_{50}$ | 57.7±2.5 [91.2%] | 22.6±2.9 | 58.3±1.6 [97.2%] | 17.6±3 |
| **F2** | 96 | Control | 0 | 0.07 ± 0.02 | 0.09 ± 0.003 | 0.2 ± 0.02 |
| | | $EC_{10}$ | 35.1±3.2 [78.0%] | 17.6 ± 2.8 | 50.2±0.4 [251%] | 15.2 ± 1.4 |
| | | $EC_{50}$ | 63.2±3.6 [105%] | 16.6 ± 4.5 | 47.6±1.8 [79.3%] | 81.3 ± 20.9 |
| **F3** | 128 | Control | 0 | 0.12±0.03 | 0.13±0.04 | 0.00 |
| | | $EC_{10}$ | 26.0±0.9 [57.7%] | 19.9±1.7 | 16.9±0.4 [84.5%] | 7.8 ± 0.6 |
| | | EC50 | 47.5±0.65 [79.1%] | 21.7±2.2 | 53.0±0.7 [88.3%] | 28.1 ± 1.8 |
| **F4** | 160 | Control | 0.68±0.13 | 0.09±0.02 | 0.04±0.04 | 0.07 ± 0.02 |
| | | $EC_{10}$ | 50.3±5.0 [112%] | 8.2±2.2 | 22.8±0.4 [114%] | 11.4 ± 1.3 |
| | | $EC_{50}$ | 52.4±0.09 [87.3%] | 13.1±1.3 | 59.5±1.02 [99.1%] | 35.6 ± 5.3 |
| **F5** | 192 | Control | 0.09±0.001 | 0.06 ± 0.01 | 0.05 ± 0.05 | 0.12±0.007 |
| | | 0 | 0.13±0.3 | 0.09 ± 0.02 | 0.0 | 0.15±0.03 |
| | | 0 | 0.09±0.1 | 0.03 ± 0.01 | 0.14 ± 0.13 | 0.41±0.07 |
| **F6** | 224 | Control | 0 | 0.05±0.003 | 0.05±0.05 | 0.05±0.009 |
| | | 0 | 0 | 0.09±0.007 | 0.0 | 0.02±0.008 |
| | | 0 | 0 | 0.06±0.01 | 0.05±0.05 | 0.04±0.008 |

**Table S3**. Detailed information regarding control performance of *Enchytraeus crypticus* at each generation of the multigenerational exposure in LUFA 2.2 soil to AgNO3 and Ag NM300K, including the survival fraction, numbers of juveniles. AV±SD: Average ± Standard Deviation. CoV: coefficient of variation. F5 and F6 were exposed to clean soil.

| Generation | Time (days) | Survival fraction (%) | CoV no. Adults (Survival) (%) | Number of juveniles (AV±SD) | CoV no. Juveniles (Reproduction) (%) |
|---|---|---|---|---|---|
| $AgNO_3$ experiment | | | | | |
| F0 | 32 | 98 ± 0.04 | 4 | 3310 ± 549 | 18 |
| F1 | 64 | 93 ± 0.05 | 5 | 5250 ± 298 | 6 |
| F2 | 96 | 90 ± 0.07 | 8 | 3061 ± 308 | 10 |
| F3 | 128 | 89 ± 0.09 | 10 | 3211 ± 345 | 10 |
| F4 | 160 | 97 ± 0.06 | 6 | 4528 ± 620 | 14 |
| F5 | 194 | 91 ± 0.17 | 20 | 4564 ± 621 | 14 |
| F6 | 224 | 97 ± 0.06 | 6 | 2700 ± 398 | 15 |
| Ag NM300K experiment | | | | | |
| F0 | 32 | 93 ± 0.06 | 7 | 3909 ± 355 | 10 |
| F1 | 64 | 94 ± 0.02 | 2 | 3200 ± 357 | 10 |
| F2 | 96 | 93 ± 0.03 | 3 | 2920 ± 251 | 9 |
| F3 | 128 | 95 ± 0.1 | 10 | 4353 ± 466 | 10 |
| F4 | 160 | 96 ± 0.04 | 5 | 4460 ± 723 | 20 |
| F5 | 194 | 94 ± 0.05 | 5 | 3640 ± 854 | 20 |
| F6 | 224 | 93 ± 0.1 | 10 | 2473 ± 428 | 20 |
